# The role of tumor microenvironment in therapeutic resistance

**DOI:** 10.18632/oncotarget.13907

**Published:** 2016-12-11

**Authors:** Beomseok Son, Sungmin Lee, HyeSook Youn, EunGi Kim, Wanyeon Kim, BuHyun Youn

**Affiliations:** ^1^ Department of Integrated Biological Science, Pusan National University, Busan 46241, Republic of Korea; ^2^ Department of Integrative Bioscience and Biotechnology, Sejong University, Seoul 05006, Republic of Korea; ^3^ Integrative Graduate Program of Ship and Offshore Plant Technology for Ocean Energy Resource, Pusan National University, Busan 46241, Republic of Korea; ^4^ Department of Biological Sciences, Pusan National University, Busan 46241, Republic of Korea

**Keywords:** tumor microenvironment, therapeutic resistance, myeloid cells, cancer-associated fibroblasts, mesenchymal stem cells

## Abstract

Cancer cells undergo unlimited progression and survival owing to activation of oncogenes. However, support of the tumor microenvironment is essential to the formation of clinically relevant tumors. Recent evidence indicates that the tumor microenvironment is a critical regulator of immune escape, progression, and distant metastasis of cancer. Moreover, the tumor microenvironment is known to be involved in acquired resistance of tumors to various therapies. Despite significant advances in chemotherapy and radiotherapy, occurrence of therapeutic resistance leads to reduced efficacy. This review highlights myeloid cells, cancer-associated fibroblasts, and mesenchymal stem cells consisting of the tumor microenvironment, as well as the relevant signaling pathways that eventually render cancer cells to be therapeutically resistant.

## INTRODUCTION

Cancer causes the most deaths among other diseases worldwide, and was responsible for 8.2 million deaths in 2012 [1]. Several key factors including tobacco and alcohol use, overweight, viral infection, and environmental pollution are known to be responsible for about 30% of cancer deaths. These factors ultimately change the molecular characteristics of cells and may initiate the uncontrolled formation of abnormal cells and protein mutations in any part of the body. Mutated epithelial growth factor receptor (EGFR), ras oncogenes, p53, and c-myc have been targets of molecularly targeted therapies [2–4]. Much progress has been made in controlling cancers over the last few decades. Breast cancer mortality rates have dropped by nearly 30% and 5-year overall survival rates of breast cancer have shown an increase of 90% over more than twenty years [5]. During the same period of time, the overall survival rates for metastatic colorectal cancer have increased from 12 to 30 months [6]. However, there are still many obstacles to overcome, including tumor cell resistance to chemotherapy and radiotherapy. For example, *FLT3* gene mutations have an internal tandem duplication of the juxta-membrane domain (FLT3/ITD), which results in chemotherapeutic resistance in acute myeloid leukemia and subsequent decreases in the progression-free survival of patients at 4 years relative to FLT3/wild-type patients (31% *versus* 55%) [7]. The tumor microenvironment includes non-cancerous cells in the tumor and proteins expressed by them that contribute to tumor growth. Increasing evidence suggests that the tumor microenvironment is a critical factor inducing cancer therapeutic resistance [8]. For example, increased matrix stiffness of hepatocellular carcinoma cells promotes resistance to chemotherapy [9]. Among various components constituting the tumor microenvironment, this review focused on myeloid, stromal, and mesenchymal stem cells (MSCs), particularly how they contribute to development of therapeutic resistance by interacting with tumor cells.

## THERAPEUTIC RESISTANCE REGULATED BY MYELOID CELLS

Bone marrow derived-myeloid cells constitute a part of the tumor microenvironment, and their cellular functions can be modulated by differentiation, which converts them to active myeloid cells. Mature myeloid cells in tumor microenvironments are reportedly involved in tumor growth, malignant progression, invasion, and therapeutic resistance [10]. There are several types of myeloid cells that function in tumor microenvironments including tumor-associated macrophages (TAMs), tumor-associated neutrophils (TANs) and myeloid-derived suppressor cells (MDSCs), each of which has unique ways to induce tumor therapeutic resistance.

TAMs, which are referred to as macrophages infiltrating into tumor tissues, are derived from monocytes and recruited to tumor tissue through expression of chemokines [11]. Activated macrophages are usually classified as M1 or M2. In the tumor microenvironment, TAMs are primarily differentiated into M2 macrophages, which have tumorigenic functions and are less cytotoxic than M1 macrophages. M2 macrophages reportedly induce therapeutic resistance *via* several mediators [11]. First, TAMs induce epithelial-to-mesenchymal transition (EMT) of tumor cells by activating the EMT signaling pathway and extracellular matrix (ECM) remodeling of the tumor microenvironment. Transforming growth factor-β (TGF-β) and tumor necrosis factor-α (TNF-α) are commonly suggested as EMT inducers secreted from TAMs [12]. Proteases such as cathepsins and matrix metalloproteinases (MMPs) are supposed to degrade the protein component of ECM, which induces EMT of cancer, invasion, and metastasis, consequently leading to therapeutic resistance [13]. For example, IL-4 is known to induce cathepsin protease activity and upregulation of Toll-like receptor 2 signaling and attenuation of glucose transporter 1/membrane type 1-MMP/MMP2 pathway are involved in tumor expansion and invasion [14–16]. TAMs-mediated tumor therapeutic resistance can be induced by angiogenesis [17]. Many studies have suggested that TAMs secreted proteins, including MMPs, plasmin, urokinase-type plasminogen activator, vascular endothelial growth factor (VEGF), interleukin (IL)-8, basic fibroblast growth factor (bFGF), thymidine phosphorylates, phosphatidylinositol-glycan biosynthesis class F protein, and gastrin-releasing peptide that can cause angiogenesis in tumor tissue [11, 18, 19]. It has been suggested that immunosuppressive factors secreted from TAMs induce therapeutic resistance. For instance, prostaglandin E2, IL-10, TGF-β, indoleamine-pyrrole 2,3-dioxygenase, chemokine C-C motif ligand (CCL) 17, CCL18, and CCL22 produced from TAMs create immunosuppressive conditions by inhibition of Th1 immune response [20–22].

Although neutrophils are a type of leukocyte, TANs residing in the tumor microenvironment are divided into anti-tumor and pro-inflammatory N1 type or tumor-progressive and immunosuppressive N2. Similar to TAMs, TANs are more likely to be polarized to the N2 type in the tumor microenvironment [23]. Many reports have indicated that TANs cause therapeutic resistance of cancers through secretion of proteins. Specifically, TANs secrete MMP2, oncostatin M, and hepatocyte growth factor (HGF), which remodel ECM and subsequently induce tumor invasion and metastasis [24–26]. They also reportedly secrete MMP9, VEGF, and Bv8, leading to increased angiogenesis [27]. Moreover, some studies have revealed that TANs increase chemoresistance through secretory factors that recruit macrophages and regulatory T cells [28]. Although the molecular mechanisms of these processes have yet to be described, these findings suggest novel pathways leading to cancer therapeutic resistance in which both TANs and TAMs are involved.

MDSCs are differentiated from myeloid precursor cells and immunosuppressive myeloid cells found in various tissues. Tumor-associated MDSCs play roles in the development of tumor therapeutic resistance through suppression of immunogenic activity and polarization of myeloid cells [29, 30]. IL-10 from MDSCs has been suggested as a major determinant of immunogenic activity in tumor microenvironments. Specifically, it was reported that IL-10 secreted from MDSCs suppressed activation of macrophages and secretion of immunogenic cytokines, including IL-6 and TNF-α from macrophages [31]. Additionally, IL-10 from MDSCs was shown to decrease maturation of dendritic cells and subsequently reduce activation of intratumoral immunity [32]. Moreover, Indu et al. demonstrated the necessity for further investigation of MDSCs because of their potential to induce resistance to doxorubicin and mephalan *via* unknown secreted soluble factors in bone marrow myeloma [33]. These findings suggested significant roles of myeloid cells and allowed us to consider targeting them to overcome the therapeutic resistance of residing tumors.

## CANCER-ASSOCIATED FIBROBLASTS (CAFS) AND TUMOR MICROENVIRONMENT

Fibroblasts form ECM, maintain tissue microenvironment and sustain cell growth in various ways. They also play critical roles in tumor microenvironments. Fibroblasts that stimulate tumor cell growth, proliferation, and metastatic conversion are known as CAFs [34]. Many studies have suggested that CAFs induce cancer progression as well as resistance to cancer therapies through secretion of proteins, exosomes, and ECM remodeling factors [35].

Because fibroblasts reside near tumor cells, they mainly affect tumor cells in a paracrine manner. It is well known that activation of the Wnt/β-catenin signaling pathway induces therapeutic resistance in many types of cancers, including ovarian cancer, non-small cell lung cancer (NSCLC), and glioblastoma [36–38]. CAFs play roles in activation of the Wnt signaling of nearby residing tumor cells. It has been reported that decreased secretory frizzled-related protein 2 secretion and increased Wnt10B and Wnt16B secretion resulted in hyperactivation of the Wnt signaling pathway [39–41]. Activation of the Wnt/β-catenin pathway in cancer cells increased expression of p-glycoprotein and ATP-binding cassette G2, and these proteins acted as a driving force for chemoresistance [42–44]. Moreover, activation of the Wnt signaling pathway reduces intracellular reactive oxygen species and induces radioresistance in cancer cells though overexpression of cyclooxygenase-2, Ku80, and aldehyde dehydrogenase, which is known to reduce radiation-induced DNA damage [45, 46].

Exposure to growth factors or chemokines/cytokines transforms resident fibroblasts into CAFs through acquirement of excessive mesenchymal properties, which is a process called mesenchymal-to-mesenchymal transition [47]. CAFs enable tumorigenesis and transformation of malignant cells into highly aggressive cancers by production of various tumor-promoting proteins. TGF-β, FGF, epidermal growth factor (EGF), and HGF induce EMT, tumor proliferation and survival, and resistance to anti-cancer agents [48]. Among these, TGF-β is the best known inducer of EMT, and occurrence of EMT is thought to make cancer cells resistant to various therapies [49]. The TGF-β-TGFBR I/II-Smad2/3 signaling pathway is activated during EMT and Snail-1/2 and Twist are master regulators of TGF-β-induced EMT [50]. Conversely, FGF, EGF, and HGF can influence tumor cells in both autocrine and paracrine fashions. Specifically, they bind to their receptors and activate proliferative signaling pathways including PI3K-AKT and mitogen-activated protein kinase [51]. Moreover, they share the downstream signaling pathways through cross-activation, which results in chemoresistance to target-specific cancer therapies. Specifically, FGF induces the expression of Snails, Twist, and Wnt ligands, and subsequently induces EMT [52]. Overexpression of Wnt ligands has also been reported to result in a feed forward cycle of the Wnt-FGF signaling pathway, which likely induces EMT and invasion by controlling SNAI1, SNAI2, ZEB1, and ZEB2 at the transcriptional or posttranslational level. Moreover, FGF signaling increases expression of DNA-dependent protein kinase and suppresses expression of pro-apoptotic Bcl-Xl and apoptosis-inducing factor, which reduces apoptosis in response to chemo- or radiotherapy [52, 53]. Although many therapeutic approaches have focused on inhibition of secretory proteins to reduce resistance of cancer cells, it is necessary to understand the crosstalk between downstream signaling pathways and functionally active factors to fundamentally overcome the occurrence of resistance [54].

Recent studies have suggested that exosomes from fibroblasts are the driving force of cancer resistance. Exosomes are lipid membranous vesicles containing large numbers of proteins, lipids, and nucleic acids that are secreted *via* exocytotic pathways from the cells. Exosomes have internalization signal receptors on the membrane that are recognized by recipient cells [35]. After exosomes are transferred into cytoplasm *via* endocytosis and phagocytosis, they can function in the recipient cells. For example, it has been reported that fibroblast-derived exosomes activated the NOTCH pathway, increased expression of NOTCH target genes, and consequently induced chemoresistance in recipient cancer cells [55]. P-glycoprotein in exosomes plays roles in activation of pro-survival signals and increases in exocytic efflux of drugs from cancer cells [55]. Furthermore, microRNAs (miRNAs) and other substances of exosomes could be transferred into the cytosol and inhibit expression of the target mRNA. MiR-21 reportedly silences apoptotic protease activating factor 1, causing paclitaxel resistance in ovarian cancer [56]. Previous studies revealed the significance of exosomes in therapeutic resistance and suggested many promising therapeutic targets. To maximize the efficacy of cancer therapy, it is necessary to understand and specifically control the resistance-inducing contents in exosomes.

ECM remodeling is another feature of CAFs-mediated modification of the tumor microenvironment. CAFs reportedly have different gene expression profiles from normal fibroblasts. Proteins such as MMPs, which are involved in ECM remodeling, show increased expression in CAFs. ECM remodeling induces epithelial plasticity, resulting in chemoresistance in glioblastoma multiforme [57]. Integrin is a membranous protein that interacts with fibronectin, collagen, and laminin and transduces signaling pathways through activation of focal adhesion kinase (FAK) and integrin-linked kinase (ILK). FAK interaction with cortactin and ILK binding with particularly interesting new cysteine-histidine-rich 1 and parvin-α affect tumor microenvironments and these complexes reduce radiotherapeutic efficacy [58]. Because ECM is a part of the tumor microenvironment regulated by CAFs, the specific molecular mechanism of ECM-induced resistance should be investigated to fulfill the therapeutic expectations.

## UNRAVELING MECHANISMS OF IMMUNOTHERAPY RESISTANCE

It is well known that the immune system recognizes cancer cells. However, tumor cells exhibit defects in antigen presentation and loss of antigenicity, which prevents the immune system from processing them. Evasion of the immune surveillance system results in the development of malignancies and patients being refractory to treatment [59]. Tumor cells also escape the immune system by altering the tumor microenvironment, namely through recruitment of immune-suppressive cells. Immuno-oncology approaches or immunotherapy refers to the restoration and harnessing of a patient’s immune surveillance system to cure their cancer. Several recent therapeutic approaches have shown promising results. For example, adoptive T cell immunotherapy led to impressive breakthroughs in treatment of patients with a variety of cancers [60]. Furthermore, therapeutic monoclonal antibodies against cytotoxic T-lymphocyte-associated antigen 4 (CTLA-4) and programmed cell death-1 to block inhibition signals received by T cells increased the long-term survival of metastatic melanoma patients [61, 62].

Although immunotherapy is a paradigm shift in the treatment of cancer patients, there are some reports of resistance to immunotherapy [63]. Intrinsic resistance is shown in patients who fail to elicit T cell responses and antitumor activity. Patients with immunodeficient virus infection, who have received transplants, or are elderly may not have a strong systemic immune response because of a decrease in their total T-cell pool [64, 65]. Moreover, many tumor antigens are also expressed in healthy cells, which would lower the avidity of T cells for these antigens [66]. In the tumor microenvironment, secretion of TGF-β and IL-10 could inhibit the function of T cells [67, 68]. Naturally acquired resistance arises from reduced sensitivity induced by ongoing immune responses. Expression of checkpoint molecules including lymphocyte activation gene 3, T cell membrane protein 3, and B and T lymphocyte attenuator is able to inhibit the activity of T cells in the tumor microenvironment [69]. Molecular determinants of immunotherapeutic resistance have also garnered research interest. For example, preclinical models of melanoma revealed that loss of PTEN promoted immune resistance, while combinational treatment with PI3K-AKT pathway inhibitors alleviated the resistance [70]. Lykken et al. found that increased galectin-1 expression was strongly correlated with CD20 immunotherapy resistance upon analysis of transcriptomic changes in lymphoma [71]. Interestingly, a recent report suggested that reprogramming of the tumor microenvironment by the oncolytic peptide LTX-315 could overcome resistance to CTLA-4 checkpoint blockade immunotherapy [72]. A deeper understanding of factors that induce immunotherapy resistance and agents that can inhibit them is expected to expand the therapeutic applications of immunotherapy.

## MSCS IN TUMOR MICROENVIRONMENT: FRIEND OR FOE

MSCs as a subgroup of bone marrow-derived cells are ubiquitous in connective tissues [73]. They can differentiate into adipocytes, osteoblasts, chondrocytes, and fibroblasts, and have multiple roles in wound repair and immune responses [74–76]. A small proportion of MSCs circulate and it has been reported that they were recruited to the tumor microenvironment following irradiation to bilateral murine 4T1 breast carcinomas [77]. MSCs release diverse growth factors and cytokines to accelerate tumor progression, angiogenesis, and metastasis [78–80].

Roodhart et al. demonstrated that MSCs induce chemotherapeutic resistance by releasing two platinum-induced fatty acids, 12-oxo-5,8,10-heptadecatrienoic acid and hexadeca-4,7,10,13-tetraenoic acid [81]. Moreover, the inflammatory environment of hepatocellular carcinoma (HCC) stimulated TGF-β expression in MSCs, which led to autophagy and chemoresistance of HCC cells [82]. MSCs residing in tumors promote cancer development by recruiting macrophages in a TNF-α-treated environment [83]. Furthermore, human MSCs-derived exosomes induced multidrug resistance of gastric cancer cells [84]. Human MSCs reportedly enhanced motility and cytokine secretion of a head and neck squamous cell carcinoma cell line [85]. Moreover, MSCs infiltrating into prostate cancer were found to increase the cancer stem cell population and metastasis through upregulation of zinc finger E-box binding homeobox 1, Snail, CXC chemokine receptor 4, and MMP9 *via* the CCL5-androgen receptor signaling pathway [86]. MSCs were also shown to influence the characteristics of the tumor microenvironment. Specifically, they activated Wnt and TGF-β signaling pathways and provided a tumor microenvironment favorable for reacquisition and maintenance of cancer stem cells [87]. Martin et al. revealed potential EMT stimulation by MSCs in the breast tumor microenvironment using a co-culture system [88]. Additionally, breast cancer cells were shown to stimulate secretion of the chemokine CCL5 from MSCs, which then enhanced the invasion of breast cancer cells and metastasis in a paracrine manner [79]. Furthermore, the MSCs-to-CAFs transition in the tumor microenvironment promoted tumor growth through the upregulation of microvascularization and the production of tumor-stimulating paracrine factors [89]. Overall, the results described above and several reviews indicate that MSCs play important roles in cancer progression and rendering cancer cells resistant to therapeutic approaches [90–93].

However, many reports have suggested the beneficial roles of MSCs in cancer therapy [94, 95]. For example, Hou et al. explored whether MSCs could inhibit survival of liver cancer cells *via* the Wnt signaling pathway [96]. Moreover, genetically modified MSCs are utilized to treat cancer through stimulation of the immune system and induction of apoptosis [97, 98]. MSCs for cell therapy and transplantation of MSCs are considered significant in stem cell therapeutics [99]. Because MSCs have the potential to exacerbate or reduce cancer risk, more elaborate conditions to use them in cancer therapies needs to be developed [100, 101].

## TUMOR HETEROGENEITY WITH SIMILAR CHARACTERISTICS TO TUMOR MICROENVIRONMENT IN RESPECT TO THERAPEUTIC RESISTANCE

Tumor cells constituting a certain type of cancer frequently have different characteristics from one another, which is called tumor heterogeneity. Tumor heterogeneity makes clinical trials complicated because intercellular communications *via* paracrine signaling or exosomes in malignancies could contribute to increased resistance to drug treatment [102, 103]. Thus, interactions between tumor cells have significant effects on clinical outcomes similar to those of tumor microenvironment; therefore, they are worth reviewing. A previous study demonstrated that a small portion of cells in glioblastoma multiforme expressing mutated EGFR were able to expand the entire tumor cell population *via* a paracrine mechanism to eventually maintain tumor heterogeneity [104]. In addition, the intercellular paracrine signaling mediated by transcription factor Pea3 among small cell lung cancer (SCLC) subclones was important in early processes of SCLC metastatic dissemination. A mouse model revealed that SCLC consists of different types of cell populations, including those overexpressing neuroendocrine markers (Elavl like RNA binding protein 4, synaptophosin, and neural cell adhesion molecule), as well as others overexpressing mesenchymal markers (nestin, vimentin, and stem cell antigen 1) [105]. In such cases, crosstalk between the two kinds of cells impacts their states. Namely, the mesenchymal cells rendered the neuroendocrine cells to be metastatic. VEGF is produced by cells that stimulate vasculogenesis and angiogenesis. Apart from its role in angiogenesis, VEGF could induce tumor progression and occurrence of drug resistance. It has been reported that some human gastric carcinoma cells express VEGF-C, and that cells treated with recombinant VEGF-C showed increased expression of placental growth factor and autocrine motility factor [106]. When VEGF-C-overexpressing cells were inoculated into the gastric walls of nude mice, tumor growth was more accelerated relative to control cells. Tumor endothelial cells were recently reported to acquire drug resistance through activation of VEGF receptor 2 in the tumor microenvironment [107]. Although a multitude of VEGF inhibitors have been tested clinically, only a few agents have been approved, and intrinsic or acquired resistance to VEGF inhibition reduced their efficacy. Studies are currently being conducted to elucidate the mechanisms responsible for resistance to VEGF [108]. In summary, the malignant characteristics of tumors may result from a small population of cells transmitting certain signals to adjacent cells.

A tumor cell might be influenced by molecules secreted from other cells [109]. Vilalta et al. suggested that granulocyte-macrophage colony stimulating factor secreted by irradiated breast tumors recruited circulating breast cancer cells, which may explain the mechanism of tumor recurrence following radiotherapy [110]. Analysis of a series of proteins secreted in response to treatment of prostate cancer therapeutic agents revealed that Wnt16B-mediated paracrine signaling was responsible for induction of cell proliferation and EMT [111]. Sun et al. determined that Wnt16B had a high secretion level, its expression was regulated by nuclear factor-κB (NF-κB), and signals were transferred in a paracrine fashion. Exosomes released from cancer cells are known to be distinct from normal cell exosomes and have clinical significance. Cancer cells release more exosomes than normal cells, and exosomes from cancer cells contain more miRNA [112]. Moreover, exosome-mediated transfer of αvβ6 integrin between cancer cells promotes prostate cancer cell migration and metastasis [113]. These findings have significance in that a marginal population containing the integrin is capable of causing adjacent cells to possess integrin-expressing phenotypes, which could facilitate cell migration and metastasis. Following analysis of mRNA and protein expression profiles of exosome vesicles, Kucharzewska et al. demonstrated that intercellular signals such as IL-8, platelet-derived growth factors, and caveolin 1 specific to hypoxic microenvironment were modulated by secreted exosome-like vesicles in glioblastoma multiforme to activate vascular cells during tumor development [114]. It has been reported that some miRNAs transferred intercellularly *via* exosomes were capable of inducing multidrug resistance, which could be reversed by treatment with β-elemene isolated from the Chinese medicinal herb Rhizoma Zedoariae [115]. Microvesicles that originate from the outward budding and fission of the plasma membrane are distinct from exosomes produced by multivesicular bodies, which are released upon fusion with the plasma membrane [116]. In fact, microvesicles are important regulators of transferring proteins and RNAs that influence tumor progression and drug resistance [117]. Secreted molecules such as exosome vesicles and microvesicles are occasionally modifiers of tumor characteristics that influence the microenvironment.

Intercellular communication within tumors could limit the benefits of radiotherapy or chemotherapy by inducing therapeutic resistance. When cancer cells were exposed to radiation, activation of NF-κB signaling occurring in the radiation-exposed cells was able to mediate bystander effects on adjacent cells, leading to radiation protection by increased expression of TNF-α, IL-1α, cMYC, and superoxide dismutase 2 [118]. Additionally, radioresistant NSCLC cells could secrete plasminogen activator inhibitor-1 and cause nearby radiosensitive NSCLC cells to be more aggressive [119]. Inhibition of caspase-3 activity and activation of AKT and ERK1/2 signaling were involved in those events. Jaiswal et al. revealed that leukemia and breast cancer cells, which have multidrug resistance, transfer nucleic acids in the form of microparticles to their drug-sensitive counterparts [120, 121]. MiRNAs conferred by microparticles affect transcription of the recipient cells, resulting in their possessing and disseminating drug-resistant phenotypes. More recently, they conducted proteome analysis of breast cancer-derived microparticles and found 120 proteins found only in drug-resistant microparticles. [122]. In a three-dimensional cell culture system in which soft sarcoma cells express higher levels of ECM-related proteins and gap junction molecules than two-dimensional systems, the cells showed elevated survival and decreased apoptosis in response to irradiation as well as doxorubicin, gemcitabine, and docetaxel treatment [123]. The results indicate that intercellular communication among cancer cells could be essential to regulating therapeutic resistance. Tumors are composed of tumor cells that have different features from each other, and their communication *via* secreted molecules may alter the tumor microenvironment and induce therapeutically resistant phenotypes.

## FUTURE PERSPECTIVES AND STRATEGIES TO OVERCOME THERAPEUTIC RESISTANCE BY MODULATING TUMOR MICROENVIRONMENT

**Figure 1:**
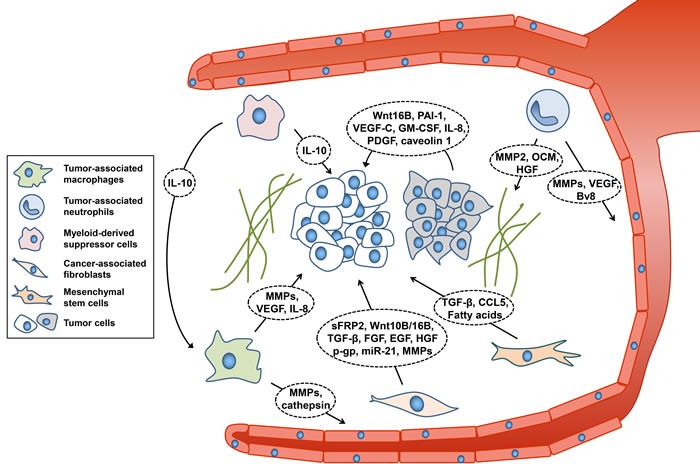
Components of tumor microenvironment affecting therapeutic resistance Cancer cells are closely associated with their microenvironment. Myeloid cells, CAFs, and MSCs secrete diverse molecules that lead to resistance to therapeutic approaches and decreased efficacy. CCL5, chemokine (C-C motif) ligand 5; EGF, epidermal growth factor; FGF, fibroblast growth factor; GM-CSF, granulocyte-macrophage colony-stimulating factor; HGF, hepatocyte growth factor; IL-8/10, Interleukin-8/10; MMPs, matrix metalloproteinases; OCM, oncostatin M; PAI-1, plasminogen activator inhibitor-1; PDGF, platelet-derived growth factor; p-gp, p-glycoprotein; sFRP2, secretory frizzled-related protein 2; TGF-β, transforming growth factor beta; VEGF, vascular endothelial growth factor.

We suggested above involvement of the tumor microenvironment and their effectors in cancer therapeutic resistance. Many types of cells contribute to the induction of therapeutic resistance through their independent mechanisms (Figure 1). For example, neighboring tumor cells definitely influence their characteristics. Intercellular communication within the tumor and its heterogeneity result in increased resistance to various therapeutic approaches. Moreover, tumor cells often utilize secreted molecules such as exosomes to communicate. It would be promising to target intratumoral interactions in anticancer therapies because they contribute significantly to the therapeutic resistance of tumor cells. β-elemene treatment was shown to inhibit transfer of multidrug resistance-associated miRNAs and thereby block intercellular communication in the tumor [115]. Moreover, inhibition of PAI-1 and interaction among NSCLC cells led to increased *in vivo* radiosensitivity [119]. Myeloid cells develop tumor therapeutic resistance through alteration of the characteristics of tumor cells, ECM remodeling and angiogenesis as discussed above. Coculture of MTLn3 cancer cells with primary bone marrow-derived macrophages isolated from cathepsin B- or S-deficient mice resulted in more impaired cancer cell invasion than coculture with macrophages from wild-type mice [14]. When combined with sorafenib (an inhibitor of tyrosine protein kinases) treatment, TANs depletion suppressed cancer growth and angiogenesis [28]. However, this discussion is not all inclusive, and other pathways of myeloid cells-induced therapeutic resistance should be investigated. As immunotherpapies are considered promising for treatment of cancer, it is more necessary than ever to identify molecular targets related to myeloid cells to overcome the current hurdles and improve therapeutic outcomes.

CAFs and MSCs are well-studied types of cells residing around tumor cells, and their importance has been highlighted in many investigations. Since therapeutic approaches targeting cancer cells have shown limitations, they could be promising targets to overcome therapeutic resistance. Paclitaxel resistance caused by stroma-derived miR-21 was attenuated by the overexpression of its target, APAF1, in mouse models [56]. Moreover, inhibitors of Raf, MEK, and ERK effectively downregulated expression of multiple drug resistance protein, multidrug resistance-related protein, and lung resistance protein [84]. Tumor microenvironment has been considered to be of great importance in various therapeutic attempts including one investigating the use of nanomedicine [124, 125]. Other parts of the tumor microenvironment have been targets of treatment [126]. Bevacizumab (Avastin) which is a variant of anti-VEGF antibody that targets endothelial cells was approved by the United States Food and Drug Administration as a therapy for metastatic colorectal cancer [127]. In terms of targeting ECM, integrin inhibitors have been suggested as therapeutic agents for cancer. For instance, cilengitide is a cyclic peptide inhibitor of integrin αvβ3 and αvβ5 for glioblastoma and other cancers [128]. Inflammatory cells-targeting PI-88 which is a heparin sulfate mimetic reduced tumorigenesis and impaired tumor growth in the later stages in a mouse model [129]. In this context, further multidisciplinary studies including bioinformatics, biochemistry, and pharmacology are required to obtain a deeper understanding of the mechanism of tumor therapeutic resistance and enhance therapeutic efficacy.

## CONCLUSIONS

Tumor microenvironment has been implicated in tumor growth, invasion, and metastasis. There has been a great deal of progress in elucidating how myeloid cells and CAFs residing in the microenvironment of tumors can affect cancer itself (Table 1). In particular, the mechanism by which tumors lose their sensitivity to conventional therapies has attracted much interest. We summarized some recent reports revealing signal cascades relevant to tumor therapeutic resistance. In addition to the aspects of tumor microenvironments described in this review, other features such as interaction of cancer cells with the ECM should be evaluated. Utilizing appropriate models that reflect characteristics of the microenvironment would extend our knowledge of cancer therapy. Moreover, it is desirable to develop therapeutic approaches targeting multiple signal pathways rather than those involved in sustaining tumor microenvironments. Investigations of tumor cells and their role in therapeutic resistance will help improve cancer treatment.

**Table 1:** A list of factors implicated in conferring therapeutic resistance to tumor cells

Source	Proteins or miRNAs	Resistant to	Molecular mechanism	References
TAMs	Cathepsin B	Paclitaxel	ECM degradation	[13]
	MMPs	Anti-angiogentic therapy	ECM degradation	[19]
	MFG-E8	Cisplatin	Activation of Stat3	[11]
	PGE2	Immunotherapy	Induction of immune suppression	[21]
	IDO	Immunotherapy	Depletion of T cell tryptophan	[22]
TANs	OSM	Anti-angiogenic therapy	Expression of VEGF	[26]
	Bv8	Anti-angiogenic therapy	Vascularization and angiogensis	[27]
MDSCs	IL-10	Immunotherapy, Sunitinib	Depletion of tumor immunity	[30]
CAFs	sFRP2	Vemurafenib	Loss of redox effector factor 1	[39]
	P-gp	Multi drug resistance	Induction of drug efflux	[44]
	miRNA-21	Paclitaxel	Targeting apoptotic protease activating factor 1	[56]
Tumor cells	VEGF-C	Verapamil	Expression of MDR1	[107]
	GM-CSF	Radiotherapy	Recruitment of circulating tumor cell	[110]
	WNT16B	Mitoxantrone, Docetaxel	Activation of Wnt pathway	[111]
	miRNA-34a; miRNA-452	Adriacin, Docetaxel	Expression of PTEN and P-gp	[115]
	PAI-1	Radiotherapy	Activation of AKT, ERK pathway	[119]

CAFs, cancer-associated fibroblasts; ECM, extracellular matrix; EMT, epithelial-to-mesenchymal transition; IDO, indoleamine-pyrrole 2,3-dioxygenase; GM-CSF, granulocyte-macrophage colony-stimulating factor; IL, interleukin; MDSCs, myeloid-derived suppressor cells; MDR1, multidrug resistance protein 1; MFG-E8; milk fat globule epidermal growth factor 8; MMP, matrix metalloproteinase; OSM, oncostatin M; PAI-1, plasminogen activator inhibitor-1; PGE2, prostaglandin E2; P-gp, P-glycoprotein; sFRP2, secreted frizzled-related sequence protein 2; TAMs, tumor-associated macrophage; TANs, tumor-associated neutrophil; VEGF, vascular endothelial growth factor.
